# A novel deleterious c.2656G>T *MSH2* germline mutation in a Pakistani family with a phenotypic overlap of hereditary breast and ovarian cancer and Lynch syndrome

**DOI:** 10.1186/s13053-016-0056-3

**Published:** 2016-07-12

**Authors:** Muhammad U. Rashid, Humaira Naeemi, Noor Muhammad, Asif Loya, Muhammed A. Yusuf, Jan Lubiński, Anna Jakubowska, Ute Hamann

**Affiliations:** Basic Sciences Research, Shaukat Khanum Memorial Cancer Hospital and Research Centre (SKMCH & RC), Lahore, Pakistan; Department of Pathology, SKMCH & RC, Lahore, Pakistan; Department of Internal Medicine, SKMCH & RC, Lahore, Pakistan; Department of Genetics and Pathology, Pomeranian Medical University, Polabska 4, 70-115 Szczecin, Poland; Molecular Genetics of Breast Cancer, German Cancer Research Center (DKFZ), Im Neuenheimer Feld 580, 69120 Heidelberg, Germany

**Keywords:** HNPCC, LS, *MSH2*, Endometrial cancer, Hereditary breast and ovarian cancer, Pakistan

## Abstract

**Background:**

Hereditary breast and ovarian cancer syndrome (HBOC) and Lynch syndrome (LS) account for a significant proportion of inherited gynecologic malignancies, mainly caused by pathogenic germline mutations in the *BRCA1* and *BRCA2* genes or in mismatch repair (*MMR*) genes, such as *MLH1* and *MSH2*. Women harboring deleterious mutations in these genes have increased life-time risks of developing a number of malignancies including ovarian cancer. Since there is a phenotypic overlap of HBOC and LS, timely identification of individuals at-risk of a particular syndrome is crucial in order to optimize cancer risk management.

**Case presentation:**

We report a novel pathogenic *MSH2* mutation, c.2656G > T, which was identified in a 67-year-old female patient with breast cancer, who had previously tested negative for a deleterious mutation in the breast cancer susceptibility genes *BRCA1*, *BRCA2*, *CHEK2* or *RAD51C*. The patient reported a personal history of endometrial cancer diagnosed at age 48, and a strong family history of breast and ovarian cancer, as well as several other malignancies within the spectrum of LS. The novel mutation was also found in the index patient’s daughter and a niece, who were diagnosed with endometrial and ovarian cancer, respectively. Breast and endometrial tumors from c.2656G > T mutation carriers showed loss of MSH2 and MSH6 protein expression. The mutation was absent in the control population.

**Conclusions:**

Our finding suggests that testing for *MMR* genes may be of benefit to *BRCA1/2* negative families with overlapping HBOC and LS phenotype in Pakistan. It is clinically significant to identify individuals harboring mutations in genes linked with a particular syndrome so that they can benefit from targeted life-saving cancer surveillance and preventive strategies.

## Background

Genetic testing for hereditary cancer has become an essential part of modern oncologic practice. It is offered to individuals with strong personal and/or family histories of cancer(s). The two most common cancer syndromes with autosomal dominant inheritance among women with gynecologic malignancies include hereditary breast and ovarian cancer (HBOC) and Lynch syndrome (LS; also known as hereditary non-polyposis colorectal cancer (HNPCC)). HBOC is mainly caused by germline mutations in the *BRCA1* and *BRCA2* genes [1], while LS is due to mutations in mismatch repair (*MMR*) genes including *MLH1*, *MSH2*, *MSH6* and *PMS2* [2]. Individuals carrying *BRCA1/2* mutations face high lifetime risks of breast and ovarian cancer [3]. Individuals with mutations in *MMR* genes have increased lifetime risks of developing colorectal, endometrial, and ovarian cancers [4–11]. Ovarian cancer is seen in both syndromes. The lifetime risks of developing breast cancer [12–16] or endometrial/colorectal carcinoma [17, 18] have also been reported to be elevated among LS- or HBOC-associated mutation carriers, respectively. Hence, there is a possible phenotypic overlap of these syndromes, which makes genetic counseling, screening, preventive and therapeutic decisions more challenging as diverse cancer risk management strategies are offered to individuals affected by HBOC or LS. Appropriate identification of individuals carrying mutations in a particular gene is an important factor in optimizing cancer risk management.

Two families, one from Italy and the other from Canada, with features of HBOC and LS have previously been described [19, 20]. In both reports double heterozygotes for *BRCA1/2* and *MLH1/MSH2* mutations were identified. Here, we report the identification of a novel single (heterozygous) deleterious c.2656G > T *MSH2* mutation in a *BRCA1/2* negative Pakistani family within the spectrum of HBOC and LS.

## Case presentation

A 67-year-old Pakistani woman of Pathan ethnicity presented with a lump in the left breast to the SKMCH & RC, Lahore, Pakistan, in October 2008. Excision biopsy revealed a grade 3 invasive ductal breast carcinoma measuring 2.8 cm in its greatest dimension (pT2) without evidence of lymph node involvement (pN0). Immunohistochemical (IHC) analyses showed that the tumor was negative for estrogen receptor (ER), progesterone receptor (PR), human epidermal growth factor receptor 2 (HER-2), MSH2 and MSH6 expression. She reported a prior diagnosis of endometrial carcinoma at age 48, for which she had undergone a total abdominal hysterectomy and bilateral salpingo-oophorectomy. In the adjuvant setting, she had received radiotherapy at University College Hospital, London, UK.

**Fig. 1:**
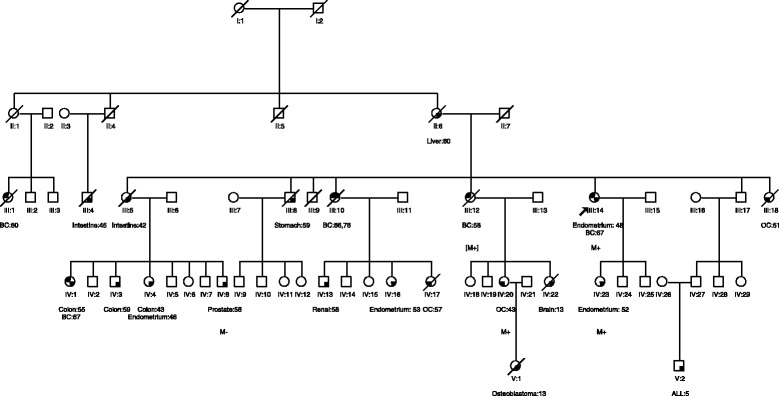
Pedigree and *MSH2* c.2656 G > T (p.Glu886*) carriers of the Pakistani cancer family 326. Circles are females, squares are males, and a diagonal slash indicates a deceased individual. Symbols with filled left upper quadrant: unilateral breast cancer. Symbols with filled upper half: bilateral breast cancer. Symbols with filled left lower quadrant: ovarian cancer. Symbols with filled right lower quadrant: cancer other than breast/ovarian cancer, the name of which is mentioned. Identification numbers of individuals are shown below the symbols. The index patient is indicated by an arrow. ALL, acute lymphoid leukemia; BC, breast cancer; OC, ovarian cancer. The numbers following these abbreviations indicate age at cancer diagnosis. M+, mutation positive. [M+], obligatory mutation carrier. M-, mutation negative

The index patient (III:14) reported an extensive family history of malignancy (Fig. 1). Her sister (III:12), cousin (III:1), and niece (IV:1) were diagnosed with unilateral breast cancer. Another sister (III:10) was diagnosed with bilateral breast cancer. Additionally, six family members had presented with gynecologic malignancies; one sister (III:18) and two nieces (IV:17, IV:20) presented with ovarian cancer; her daughter (IV:23) and two nieces (IV:4, IV:16) were diagnosed with endometrial cancer. Another sister (III:5) and one maternal cousin (III:4) had previously been diagnosed with intestinal cancer, and three descendants (IV:1, IV:3, IV:4) of that sister were affected by colon cancer. The index patient reported several other family members with various malignancies including her mother (II:6) with liver cancer, a brother (III:8) with stomach cancer, one nephew (IV:13) with renal cancer and another (IV:8) with prostate cancer, and one niece (IV:22), one grandniece (V:1) and one grandnephew (V:2) with childhood malignancies including brain tumor, osteoblastoma and leukemia, respectively. The cancer diagnoses of the index patient (III:14), her daughter (IV:23) and one niece (IV:20) were confirmed by review of medical records and/or pathological reports. All other cancer diagnoses were self-reported or reported by other family members.

Given the strong family history of cancer, the patient was referred to SKMCH & RC for genetic counseling and risk assessment and was enrolled in the study after obtaining written informed consent. The study was approved by the ethical review board of SKMCH & RC. Due to the presence of multiple breast and ovarian cancers in this family, the preliminary diagnosis was HBOC syndrome [1]. The patient had previously tested negative for deleterious small-range *BRCA1/2* mutations and large genomic rearrangements (M. U. Rashid, unpublished data) using denaturing high performance liquid chromatography (DHPLC) analysis followed by DNA sequence analysis of variant fragments and multiplex ligation-dependent probe amplification (MLPA) as described [21, 22]. The patient had also tested negative for mutations in *CHEK2* [23] and *RAD51C* [24], implying the involvement of other gene(s) contributing to disease risk in this family.

This family also fulfilled the recognized criteria for suspected LS [25] (Fig. 1): that includes (i) among first degree relatives of a colorectal cancer patient (or in himself) at least 1 colorectal cancer, cancer of the endometrium, small bowel or urinary tract; (ii) at least one of the above cancers diagnosed under age 50; and (iii) familial adenomatous polyposis excluded [25]. The index case was screened for germline mutations in the *MLH1, MSH2* and *MSH6* genes using DHPLC and DNA sequence analysis as described elsewhere [26]. A novel disease-causative heterozygous nonsense mutation in exon 16 of *MSH2*, c.2656G > T (p.Glu886*), was identified.

Genetic *MSH2* testing was offered to other affected family members. The mutation was also detected in the index patient’s daughter (IV:23) who was diagnosed with endometrial cancer, and one niece (IV:20) affected by ovarian cancer. Her nephew (IV:8), who was diagnosed with prostate cancer, tested negative for this mutation. The mutation was also not identified in 100 healthy female controls.

## Discussion

We report the identification of a deleterious *MSH2* germline mutation in a Pakistani family with a strong family history of malignancy (6 breast cancers, 3 ovarian cancers, 4 endometrial carcinomas, 3 colon cancers, 2 intestinal cancers) within the spectrum of HBOC and LS, who tested negative for *BRCA1/2* mutations. Our data highlight the clinical implications of this finding, specifically with respect to genetic counseling, screening, and prophylaxis of mutation carriers in such families.

The index patient harboring the *MSH2* mutation was affected by endometrial cancer at age 48 and breast cancer at age 67. The occurrence of breast cancer following endometrial cancer is in line with data from Win and colleagues, who showed that women with LS have an increased risk of breast cancer after endometrial cancer [27]. The mutation, c.2656G > T (p.Glu886*), located in exon 16 of the *MSH2* gene, is novel and has not been previously reported in several large variant databases (Exome Aggregation Consortium (ExAC), http://exac.broadinstitute.org/; Exome Sequence Project (ESP), http://evs.gs.washington.edu/EVS/; Human Gene Mutation Database (HGMD), http://www.hgmd.cf.ac.uk/ac/index.php); Leiden Open Variation Database (LOVD), http://chromium.liacs.nl/LOVD2/colon_cancer/, Mismatch Repair Genes Variant Database (MMRGVD), http://www.med.mun.ca/mmrvariants/ or Universal Mutation Database (UMD), http://www.umd.be/ (by May 2016). It is likely to be pathogenic as it generates a premature termination codon resulting in a truncated protein, with loss of 49 C-terminal amino acid residues, a region involved in MSH2 homodimer formation and MSH3/MSH6 interaction [28]. The location of the mutation is in a region of high sequence conservation among humans, grivets, mice and cows and it’s absence in 100 healthy controls suggests that it is associated with the disease. Several other pathogenic mutations in this region have previously been described [29, 30] (LOVD, http://chromium.liacs.nl/LOVD2/colon_cancer/; UMD database http://www.umd.be/). Moreover, the c.2656G > T mutation segregated with the disease as it was identified in the index patient’s daughter, who had herself suffered from endometrial cancer at age 52 and in a niece affected by ovarian cancer at age 43. The latter probably inherited the mutation from her deceased mother, who had been diagnosed with breast cancer at age 58, suggesting that this mutation may predispose to breast cancer. Association of *MMR* gene mutations with breast cancer has previously been observed in various studies among Australian, Brazilian, Danish, Dutch, and US populations [12–16]. Recently, a four-fold increase in breast cancer risk was reported in *MMR* gene mutation carriers from Australia, New Zealand, Canada, and the United States compared to the general population [31]. In contrast, initial studies among Finnish and US populations showed no association [4, 32]. The variation in observed data may be due to population heterogeneity or involvement of other genetic and/or non-genetic risk factors.

The breast tumor associated with the pathogenic *MSH2* mutation in our index patient was negative for ER, PR and HER2 expression, also known as triple-negative breast cancer (TNBC). This observation is in agreement with previous findings that breast tumors linked with mutations in *MMR* genes are primarily negative for ER and PR expression [33]. However, *BRCA1* associated breast tumors are also reported to significantly display TNBC [34], so that TNBC phenotype cannot be utilized to distinguish between *BRCA1* and *MMR* gene mutation carriers. The index patient’s breast tumor was also negative for MSH2 and MSH6 protein expression (data not shown). This finding suggests that the *MSH2* germline mutation, in addition to endometrial cancer, may also predispose to breast cancer in this family. Given the controversy as to whether breast cancer is a part of LS or not, in the literature, large prospective studies will help to clarify this issue.

Patients with LS have increased lifetime risks of developing ovarian, endometrial, and colorectal cancers, ranging from 6.7–13.5 %, 31.5–62 %, and 50–80 %, respectively [4–6, 9, 11]. In the family reported here, the *MSH2* mutation was found to co-segregate with ovarian and endometrial cancer. We were unable to study co-segregation of this mutation in the colon/intestinal cancer cases, since they were either deceased or did not agree to participate in this study. The mean age of colon/intestinal cancer diagnosis in this family was 48.8 years, which is similar to the mean age of 44.8 years previously reported in Caucasians with *MSH2* mutations [35].

Previously, bi-allelic germline mutations in *MMR* genes have been reported with a rare constitutional MMR-deficiency syndrome, characterised by a broad spectrum of childhood onset malignancies mostly among individuals with a history of parental consangunity [36, 37]. Among children with this syndrome, *MSH2* mutations are less common, while *PMS2* mutations are most commonly found [37]. Three paediatric cancers (leukemia, brain tumor, and osteoblastoma) were also observed in our study, with unknown parental consanguinity status. Due to the lack of DNA samples, none of these individuals (IV:22, V:1 and V:2) nor their parents (III:13, IV:21, IV:26 and IV:27) could be tested. It is unlikely that the childhood malignancies in the Pakistani family are linked with the *MSH2* mutation, as manifestations of LS in the parents of these children were not observed. Hence, the role of *PMS2* or other genes known to be involved in these childhood malignancies cannot be excluded.

Different sets of clinical criteria for identifying patients at high risk of LS include the stringent Amsterdam I/II criteria [38, 39], which are based on a family history of at least three relatives with histologically verified colorectal cancer/cancers linked with LS, respectively, and the less stringent Bethesda guidelines, later updated to the revised Bethesda guidelines [40, 41]. The revised Bethesda guidelines, based on clinicopathologic parameters, have been developed to identify high-risk patients by evaluation of microsatellite instability (MSI) and/or IHC testing of their tumors. Unfortunately, this strategy could not be applied to the Pakistani index patient due to restraints of normal/tumor tissue. However, the Pakistani family fulfilled the less stringent criteria of suspected LS [25], which is based on a family history of only two LS-linked cancers. Our finding support the notion that the suspected LS criteria may be useful for the identification of Pakistani families [25, 42, 43].

To our knowledge, this is the first Asian family with a history of cancer within the spectrum of HBOC and LS, in which a novel, single heterozygous mutation in a *MMR* gene was identified. Different findings have been reported in two other studies among two families, one from Italy and another from Canada, which have a similar phenotype, but different genotypes, with double heterozygous *BRCA1/2* and *MMR* gene mutations, were identified [19, 20], which may confer an altered risk. Women with double heterozygous *BRCA1/2* and *MMR* gene mutations were younger at time of diagnosis of breast cancer (range 32–46) than the single heterozygous Pakistani patient(s) (ages: 58 (obligatory carrier), 67) described in this report. The Pakistani patient did not harbor a deleterious mutation in *BRCA1/2* and also tested negative for mutations in *CHEK2* [23], *RAD51C* [24], and *PALB2* (M. U. Rashid, unpublished data). We cannot exclude the possibility that a mutation in yet another breast cancer susceptibility gene exists, which was not analyzed. While the mean age of breast cancer diagnosis in the Pakistani population is 47 years (range 18–90) [44], Pakistani patients with *BRCA1, CHEK2,* or *RAD51C* mutations presented with breast cancer at a mean age of 31 years (range 22–49), 41.5 years (range 30–53), and 50 years (range 49–51), respectively [21, 23, 24]. In the current pedigree, the mean age of breast cancer onset of 65.7 years (range 58–76) was higher, suggesting the involvement of other susceptibility gene(s). The deleterious mutation in the *MSH2* gene reported here may predispose to breast cancer in this family, since it was detected in one family member diagnosed with breast cancer at age 67, while another family member diagnosed with breast cancer at age 58 is likely to be an obligatory carrier, who passed the mutation on to her daughter. The co-segregation of the mutation with breast cancer could not be investigated further, because two relatives were deceased and another refused to participate. Therefore it may still be possible that the overrepresentation of breast cancer in this family is by chance, considering that breast cancer is the most common invasive malignancy in Pakistani women (Globocan 2012; http://globocan.iarc.fr/).

## Conclusions

Our findings suggest that *MMR* gene testing may be beneficial to *BRCA1/2* negative families presenting with clinical features and a pedigree chart suggestive of HBOC syndrome, especially if they report other LS-associated cancer(s). It is clinically important to identify individuals with LS, so that they can benefit from targeted life-saving cancer surveillance strategies.

## Abbreviations

DHPLC, denaturing high-performance liquid chromatography; ER, estrogen receptor; HBOC, hereditary breast and ovarian cancer; HER2, human epidermal growth factor receptor 2; HNPCC, hereditary nonpolyposis colorectal cancer; IHC, Immunohistochemical; LS, lynch syndrome; MLPA, multiplex ligation-dependent probe amplification; *MMR*, mismatch repair; MSI, microsatellite instability; PR, progesterone receptor; SKMCH & RC, Shaukat Khanum Memorial Cancer Hospital and Research Centre, Lahore, Pakistan; TNBC, triple-negative breast cancer

## References

[1] Apostolou P, Fostira F (2013). Hereditary breast cancer: the era of new susceptibility genes. Biomed Res Int.

[2] Jansen M, Menko FH, Brosens LA, Giardiello FM, Offerhaus GJ (2014). Establishing a clinical and molecular diagnosis for hereditary colorectal cancer syndromes: Present tense, future perfect?. Gastrointest Endosc.

[3] Ford D, Easton DF, Stratton M, Narod S, Goldgar D, Devilee P, Bishop DT, Weber B, Lenoir G, Chang-Claude J (1998). Genetic heterogeneity and penetrance analysis of the BRCA1 and BRCA2 genes in breast cancer families. Am J Hum Genet.

[4] Aarnio M, Sankila R, Pukkala E, Salovaara R, Aaltonen LA, la CA D, Peltomaki P, Mecklin JP, Jarvinen HJ (1999). Cancer risk in mutation carriers of DNA-mismatch-repair genes. Int J Cancer.

[5] Hampel H, Stephens JA, Pukkala E, Sankila R, Aaltonen LA, Mecklin JP, la CA D (2005). Cancer risk in hereditary nonpolyposis colorectal cancer syndrome: later age of onset. Gastroenterology.

[6] Quehenberger F, Vasen HF, van Houwelingen HC (2005). Risk of colorectal and endometrial cancer for carriers of mutations of the hMLH1 and hMSH2 gene: correction for ascertainment. J Med Genet.

[7] Muller A, Schackert HK, Lange B, Ruschoff J, Fuzesi L, Willert J, Burfeind P, Shah P, Becker H, Epplen JT (2006). A novel MSH2 germline mutation in homozygous state in two brothers with colorectal cancers diagnosed at the age of 11 and 12 years. Am J Med Genet A.

[8] Lancaster JM, Powell CB, Kauff ND, Cass I, Chen LM, Lu KH, Mutch DG, Berchuck A, Karlan BY, Herzog TJ (2007). Society of Gynecologic Oncologists Education Committee statement on risk assessment for inherited gynecologic cancer predispositions. Gynecol Oncol.

[9] Watson P, Vasen HF, Mecklin JP, Bernstein I, Aarnio M, Jarvinen HJ, Myrhoj T, Sunde L, Wijnen JT, Lynch HT (2008). The risk of extra-colonic, extra-endometrial cancer in the Lynch syndrome. Int J Cancer.

[10] Geary J, Sasieni P, Houlston R, Izatt L, Eeles R, Payne SJ, Fisher S, Hodgson SV (2008). Gene-related cancer spectrum in families with hereditary non-polyposis colorectal cancer (HNPCC). Fam Cancer.

[11] Brosens LA, Offerhaus GJ, Giardiello FM (2015). Hereditary Colorectal Cancer: Genetics and Screening. Surg Clin North Am.

[12] Boyd J, Rhei E, Federici MG, Borgen PI, Watson P, Franklin B, Karr B, Lynch J, Lemon SJ, Lynch HT (1999). Male breast cancer in the hereditary nonpolyposis colorectal cancer syndrome. Breast Cancer Res Treat.

[13] Scott RJ, McPhillips M, Meldrum CJ, Fitzgerald PE, Adams K, Spigelman AD, Du SD, Tucker K, Kirk J (2001). Hereditary nonpolyposis colorectal cancer in 95 families: differences and similarities between mutation-positive and mutation-negative kindreds189. Am J Hum Genet.

[14] de Leeuw WJ, Van PM, Tollenaar RA, Cornelisse CJ, Vasen HF, Morreau H (2003). Correspondence re: A. Muller et al., Exclusion of breast cancer as an integral tumor of hereditary nonpolyposis colorectal cancer. Cancer Res., 62: 1014–1019, 2002.. Cancer Res.

[15] Jensen UB, Sunde L, Timshel S, Halvarsson B, Nissen A, Bernstein I, Nilbert M (2010). Mismatch repair defective breast cancer in the hereditary nonpolyposis colorectal cancer syndrome. Breast Cancer Res Treat.

[16] da Silva FC, de Oliveira LP, Santos EM, Nakagawa WT, Aguiar JS, Valentin MD, Rossi BM, De Oliveira FF (2010). Frequency of extracolonic tumors in Brazilian families with Lynch syndrome: analysis of a hereditary colorectal cancer institutional registry. Fam Cancer.

[17] Lavie O, Ben-Arie A, Segev Y, Faro J, Barak F, Haya N, Auslender R, Gemer O (2010). BRCA germline mutations in women with uterine serous carcinoma--still a debate. Int J Gynecol Cancer.

[18] Phelan CM, Iqbal J, Lynch HT, Lubinski J, Gronwald J, Moller P, Ghadirian P, Foulkes WD, Armel S, Eisen A (2014). Incidence of colorectal cancer in BRCA1 and BRCA2 mutation carriers: results from a follow-up study. Br J Cancer.

[19] Thiffault I, Hamel N, Pal T, McVety S, Marcus VA, Farber D, Cowie S, Deschenes J, Meschino W, Odefrey F (2004). Germline truncating mutations in both MSH2 and BRCA2 in a single kindred. Br J Cancer.

[20] Pedroni M, Di GC, Cortesi L, Reggiani BL, Magnani G, Simone ML, Medici V, Priore OC, Marino M, de Ponz LM (2014). Double heterozygosity for BRCA1 and hMLH1 gene mutations in a 46-year-old woman with five primary tumors. Tech Coloproctol.

[21] Rashid MU, Zaidi A, Torres D, Sultan F, Benner A, Naqvi B, Shakoori AR, Seidel-Renkert A, Farooq H, Narod S (2006). Prevalence of BRCA1 and BRCA2 mutations in Pakistani breast and ovarian cancer patients. Int J Cancer.

[22] Ticha I, Kleibl Z, Stribrna J, Kotlas J, Zimovjanova M, Mateju M, Zikan M, Pohlreich P (2010). Screening for genomic rearrangements in BRCA1 and BRCA2 genes in Czech high-risk breast/ovarian cancer patients: high proportion of population specific alterations in BRCA1 gene. Breast Cancer Res Treat.

[23] Rashid MU, Muhammad N, Faisal S, Amin A, Hamann U (2013). Constitutional CHEK2 mutations are infrequent in early-onset and familial breast/ovarian cancer patients from Pakistan. BMC Cancer.

[24] Rashid MU, Muhammad N, Faisal S, Amin A, Hamann U (2014). Deleterious RAD51C germline mutations rarely predispose to breast and ovarian cancer in Pakistan. Breast Cancer Res Treat.

[25] Kladny J, Lubinski J (2008). Lynch syndrome (HNPCC). Hered Cancer Clin Pract.

[26] Kurzawski G, Safranow K, Suchy J, Chlubek D, Scott RJ, Lubinski J (2002). Mutation analysis of MLH1 and MSH2 genes performed by denaturing high-performance liquid chromatography. J Biochem Biophys Methods.

[27] Win AK, Lindor NM, Winship I, Tucker KM, Buchanan DD, Young JP, Rosty C, Leggett B, Giles GG, Goldblatt J (2013). Risks of colorectal and other cancers after endometrial cancer for women with Lynch syndrome. J Natl Cancer Inst.

[28] Guerrette S, Wilson T, Gradia S, Fishel R (1998). Interactions of human hMSH2 with hMSH3 and hMSH2 with hMSH6: examination of mutations found in hereditary nonpolyposis colorectal cancer. Mol Cell Biol.

[29] Percesepe A, Borghi F, Menigatti M, Losi L, Foroni M, Di GC, Rossi G, Pedroni M, Sala E, Vaccina F (2001). Molecular screening for hereditary nonpolyposis colorectal cancer: a prospective, population-based study. J Clin Oncol.

[30] Swensen J, Lewis CM, Cannon-Albright LA (1997). Identification of a one-base germline deletion (codon 888 del C) and an intron splice acceptor site polymorphism in hMSH2. Hum Mutat.

[31] Win AK, Young JP, Lindor NM, Tucker KM, Ahnen DJ, Young GP, Buchanan DD, Clendenning M, Giles GG, Winship I (2012). Colorectal and other cancer risks for carriers and noncarriers from families with a DNA mismatch repair gene mutation: a prospective cohort study. J Clin Oncol.

[32] Watson P, Lynch HT (1993). Extracolonic cancer in hereditary nonpolyposis colorectal cancer. Cancer.

[33] Walsh MD, Buchanan DD, Cummings MC, Pearson SA, Arnold ST, Clendenning M, Walters R, McKeone DM, Spurdle AB, Hopper JL (2010). Lynch syndrome-associated breast cancers: clinicopathologic characteristics of a case series from the colon cancer family registry. Clin Cancer Res.

[34] Reis-Filho JS, Tutt AN (2008). Triple negative tumours: a critical review. Histopathology.

[35] Kastrinos F, Stoffel EM, Balmana J, Steyerberg EW, Mercado R, Syngal S (2008). Phenotype comparison of MLH1 and MSH2 mutation carriers in a cohort of 1,914 individuals undergoing clinical genetic testing in the United States. Cancer Epidemiol Biomarkers Prev.

[36] Lavoine N, Colas C, Muleris M, Bodo S, Duval A, Entz-Werle N, Coulet F, Cabaret O, Andreiuolo F, Charpy C (2015). Constitutional mismatch repair deficiency syndrome: clinical description in a French cohort. J Med Genet.

[37] Wimmer K, Kratz CP, Vasen HF, Caron O, Colas C, Entz-Werle N, Gerdes AM, Goldberg Y, Ilencikova D, Muleris M (2014). Diagnostic criteria for constitutional mismatch repair deficiency syndrome: suggestions of the European consortium ’care for CMMRD’ (C4CMMRD). J Med Genet.

[38] Vasen HF, Watson P, Mecklin JP, Lynch HT (1999). New clinical criteria for hereditary nonpolyposis colorectal cancer (HNPCC, Lynch syndrome) proposed by the International Collaborative group on HNPCC. Gastroenterology.

[39] Vasen HF, Mecklin JP, Khan PM, Lynch HT (1991). The International Collaborative Group on Hereditary Non-Polyposis Colorectal Cancer (ICG-HNPCC). Dis Colon Rectum.

[40] Umar A, Boland CR, Terdiman JP, Syngal S, la CA D, Ruschoff J, Fishel R, Lindor NM, Burgart LJ, Hamelin R (2004). Revised Bethesda Guidelines for hereditary nonpolyposis colorectal cancer (Lynch syndrome) and microsatellite instability. J Natl Cancer Inst.

[41] Rodriguez-Bigas MA, Boland CR, Hamilton SR, Henson DE, Jass JR, Khan PM, Lynch H, Perucho M, Smyrk T, Sobin L (1997). A National Cancer Institute Workshop on Hereditary Nonpolyposis Colorectal Cancer Syndrome: meeting highlights and Bethesda guidelines. J Natl Cancer Inst.

[42] Park JG, Vasen HF, Park KJ, Peltomaki P, de Ponz LM, Rodriguez-Bigas MA, Lubinski J, Beck NE, Bisgaard ML, Miyaki M (1999). Suspected hereditary nonpolyposis colorectal cancer: International Collaborative Group on Hereditary Non-Polyposis Colorectal Cancer (ICG-HNPCC) criteria and results of genetic diagnosis. Dis Colon Rectum.

[43] Park JG, Vasen HF, Park YJ, Park KJ, Peltomaki P, de Leon MP, Rodriguez-Bigas MA, Lubinski J, Beck NE, Bisgaard ML (2002). Suspected HNPCC and Amsterdam criteria II: evaluation of mutation detection rate, an international collaborative study. Int J Colorectal Dis.

[44] Khokher S, Qureshi MU, Riaz M, Akhtar N, Saleem A (2012). Clinicopathologic profile of breast cancer patients in Pakistan: ten years data of a local cancer hospital. Asian Pac J Cancer Prev.

